# When the Cecum Hides a Sarcoma: A Rare Case of Undifferentiated Malignancy

**DOI:** 10.7759/cureus.104908

**Published:** 2026-03-09

**Authors:** Soufia El Ouardani, Hanan Bailal, Mohamed Mouhoub, Hind Chibani, Sami Aziz Brahmi, Said Afqir

**Affiliations:** 1 Medical Oncology, Mohammed VI University Hospital, Oujda, MAR; 2 Medical Oncology, Faculty of Medicine and Pharmacy of Oujda, Mohamed First University, Oujda, MAR; 3 Pathology, Centre de Diagnostic Anatomopathologique Dr. Mouhoub, Oujda, MAR

**Keywords:** chemotherapy, gastrointestinal tumor, peritoneal carcinomatosis, surgery, undifferentiated sarcoma

## Abstract

Primary undifferentiated sarcoma of the cecum is an exceptionally rare and aggressive mesenchymal tumor, often difficult to diagnose due to its nonspecific presentation and overlap with more common colonic malignancies. We report the case of a 66-year-old patient presenting with right iliac fossa pain and bowel transit disturbances. Imaging revealed a large cecal mass without distant metastasis. The patient underwent surgical resection. Histopathological and immunohistochemical examination confirmed a primary undifferentiated sarcoma of the cecum. Postoperative management was discussed in a multidisciplinary setting. Through this case, we highlight the diagnostic challenges and therapeutic considerations of this rare entity.

## Introduction

Primary sarcomas of the colon are extremely rare, accounting for approximately 0.1% of all colorectal malignancies [1]. Undifferentiated sarcomas, typically seen in extremities or retroperitoneum, are exceptionally uncommon in the gastrointestinal tract [2]. These tumors are mesenchymal in origin and may arise from various mesenchymal components of the intestinal wall, including smooth muscle and other connective tissue elements [3]. Their clinical presentation is nonspecific, including abdominal pain, bleeding, or weight loss, often mimicking adenocarcinomas [4].

This rarity and overlapping symptoms make preoperative diagnosis particularly challenging [1]. Imaging may reveal a mass, but definitive diagnosis requires histopathology and immunohistochemistry [5]. Early recognition is crucial, as these tumors are aggressive and may present at an advanced stage [3]. The overall five-year survival for colorectal sarcomas has been reported to be approximately 50.3%, increasing to 60.8% among patients who underwent surgical resection. Poorer prognosis is associated with the presence of distant metastases, regional disease spread, and high-grade tumors, according to a United States population-based analysis of colorectal leiomyosarcoma [6].

Despite limited literature, case reports contribute valuable insights into their behavior and management [4,7]. Here, we report a rare case of undifferentiated sarcoma of the cecum, highlighting diagnostic difficulties and treatment considerations.

## Case presentation

A 66-year-old male patient with a past medical history of well-controlled hypertension presented with a two-month history of progressively worsening right lower quadrant abdominal pain associated with altered bowel habits and intermittent episodes of lower gastrointestinal bleeding. He also reported marked asthenia, anorexia, and an unintentional weight loss of approximately 6 kg. There was no history of previous abdominal surgery or known malignancy.

On physical examination, the patient appeared pale, and abdominal palpation revealed a firm, tender mass in the right iliac fossa without signs of peritonitis. Laboratory investigations demonstrated microcytic hypochromic anemia: hemoglobin 9.2 g/dL(reference range in men: 13-17 g/dL), and elevated C-reactive protein levels: 70 mg/L (reference range: <5 mg/L).

**Figure 1:**
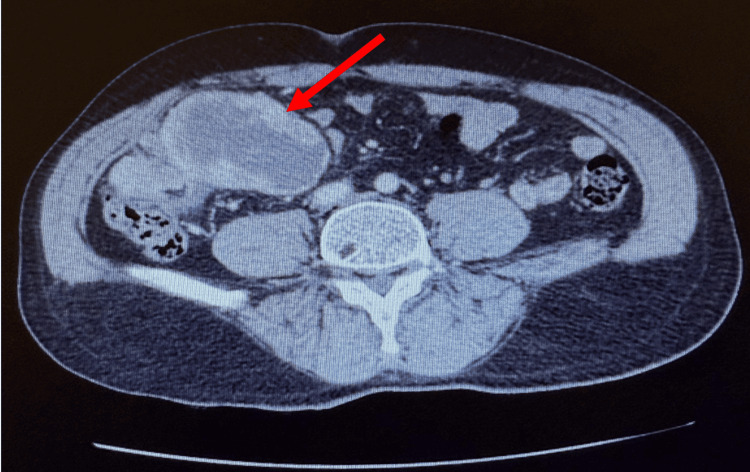
Axial contrast-enhanced CT scan of the abdomen. A large heterogeneous mass in the right iliac fossa (red arrow), without evidence of locoregional invasion and with preserved surrounding fat planes. The lesion measured approximately 105 × 70 × 100 mm and appeared solid, irregular, and well circumscribed.

Preoperative CT imaging showed a well-circumscribed cecal mass without locoregional invasion or suspicious lymphadenopathy, which supported the preoperative suspicion of a gastrointestinal stromal tumor (GIST) (Figure 1). The patient underwent an exploratory laparotomy with right hemicolectomy and en bloc resection of the tumor. Therefore, a formal lymph node dissection was not performed, consistent with standard management of GIST and sarcomas that rarely metastasize via lymphatics.

**Figure 2:**
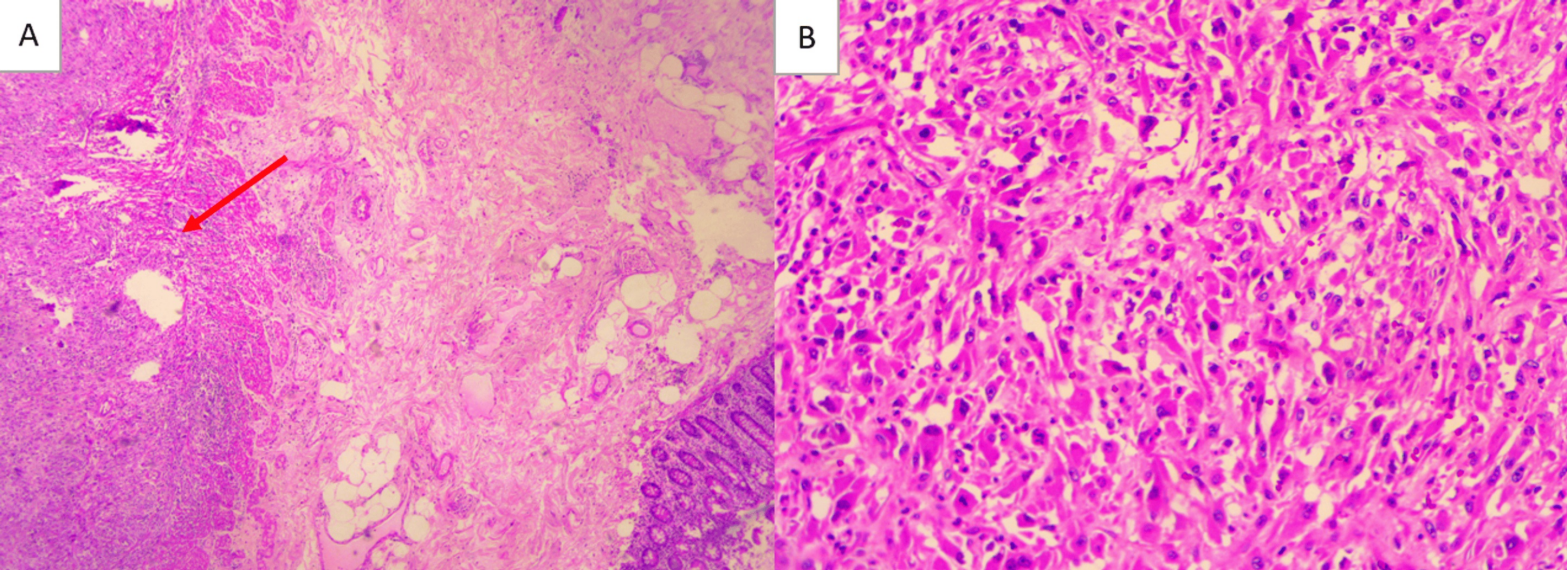
Photomicrograph of the tumor. (A) Histological examination showed a preserved mucosa with intact intestinal glands, while the tumor arises from the muscularis propria (red arrow) (H&E, ×5) (B) The tumor consists of highly atypical cells, mostly spindle-shaped, with eosinophilic cytoplasm and occasional globular or rhabdoid forms. The nuclei are centrally located, markedly atypical, with prominent nucleoli, and some cells are multinucleated giant forms. Numerous atypical mitotic figures are present, highlighting the high-grade malignant nature of the neoplasm (H&E, ×20)

**Figure 3:**
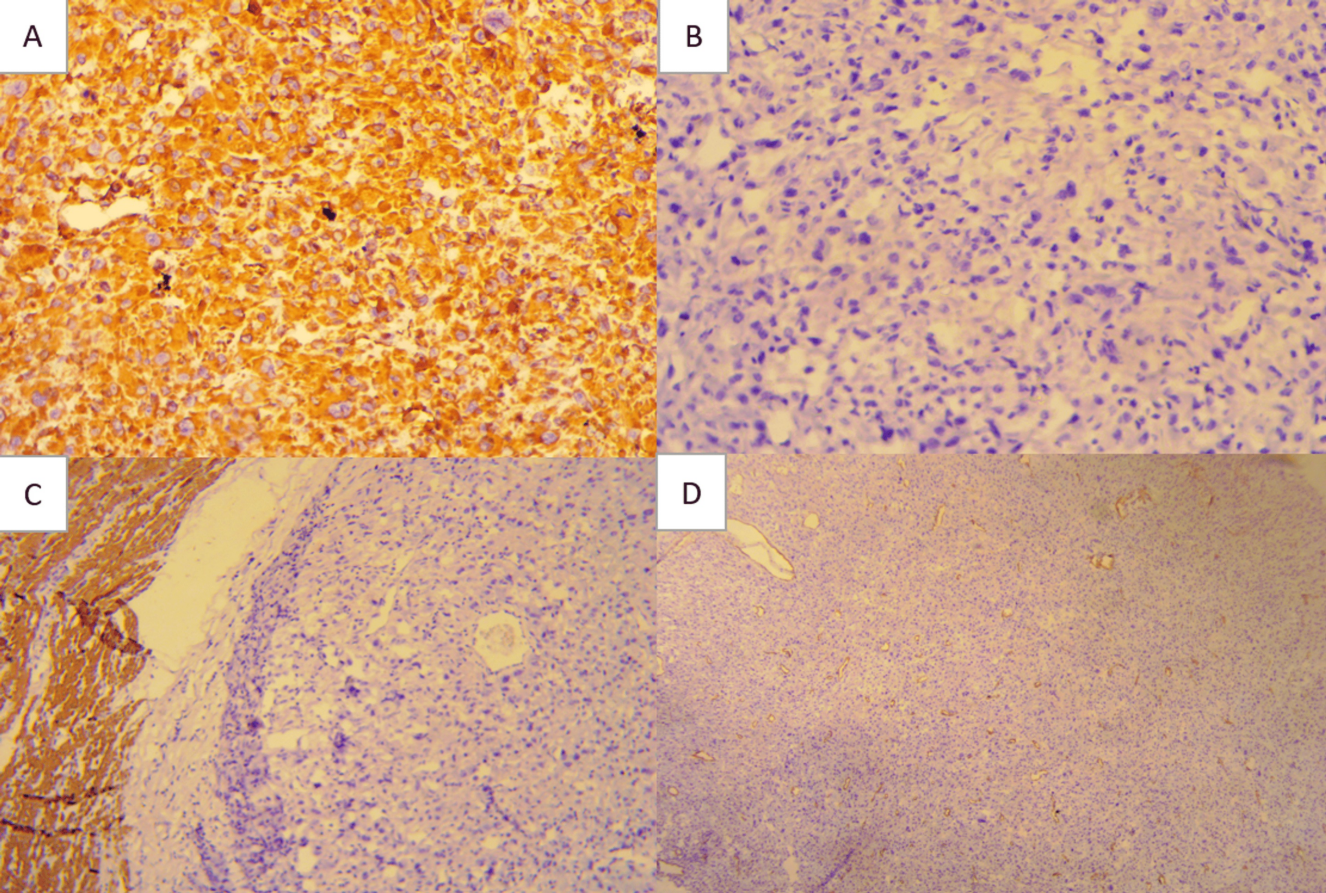
Immunohistochemical analysis of tumor tissue. (A) Positive staining of tumor cells by anti-vimentin (DAB, x20); (B) Tumor cells lacked expression of CD117 (DAB, x20); (C) Tumor cells lacked expression of Desmin (DAB, x10); (D) Absence of AML expression (DAB, x5) DAB: 3,3′-diaminobenzidine; AML: acute myeloid leukemia

Histopathological analysis revealed a high-grade undifferentiated sarcoma of the cecum, exhibiting marked cellular pleomorphism and a high mitotic index (Figure 2). Extensive tumor necrosis was present. The Ki-67 proliferation index was 40%, and the tumor was graded as Fédération Nationale des Centres de Lutte Contre le Cancer (FNCLCC) Grade 3 (high grade). The deep surgical margin was involved by tumor, whereas all other margins were free of neoplastic infiltration. Immunohistochemically, the tumor cells were strongly positive for vimentin and negative for cytokeratin (CK AE1/AE3), cluster of differentiation 117 (CD117, c-Kit), discovered on GIST-1 (DOG-1), acute myeloid leukemia marker (AML), cyclin-dependent kinase 4 (CDK4), desmin, myogenin, CD34, S-100, CD10, and melan-A (Figure 3).

**Figure 4:**
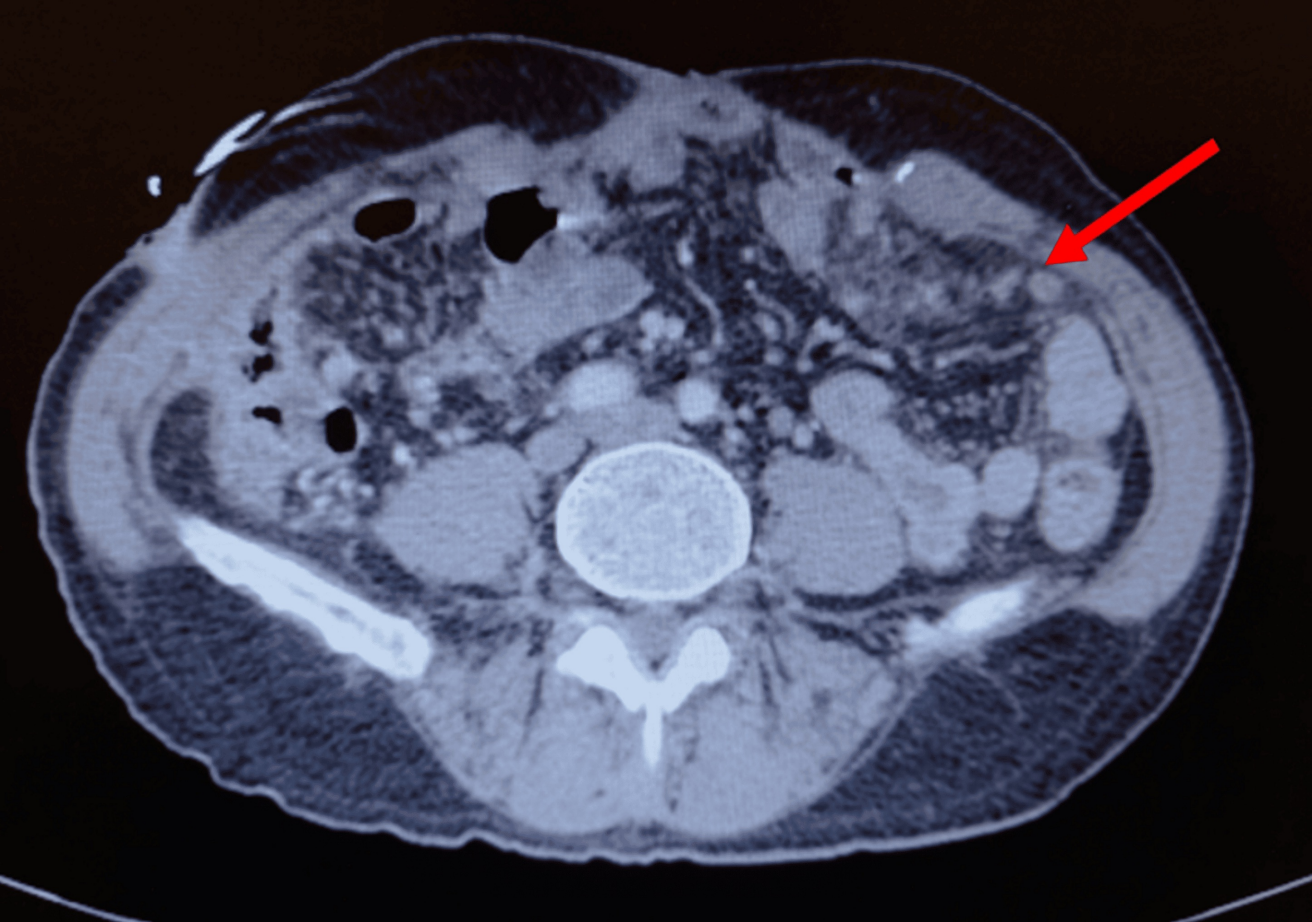
Contrast-enhanced CT scan (axial view) of the abdomen six weeks after surgery showing diffuse peritoneal carcinomatosis (red arrow)

In the postoperative period, the patient’s clinical condition progressively deteriorated. A CT scan performed six weeks after surgery demonstrated diffuse peritoneal carcinomatosis consistent with disease progression, without hepatic or pulmonary metastases (Figure 4). Palliative systemic chemotherapy based on doxorubicin and ifosfamide was initiated.

However, the patient’s general condition further declined (Eastern Cooperative Oncology Group (ECOG) Performance status was 3) after one cycle of chemotherapy, leading to treatment discontinuation. He was subsequently referred to palliative care and later passed away.

## Discussion

Sarcomas are rare malignant tumors of mesenchymal origin, accounting for less than 1% of adult malignancies [1,8]. Within the gastrointestinal tract, they represent approximately 0.1-1% of digestive cancers, with the stomach and small intestine being more frequently involved [1,6]. Primary colorectal sarcomas are particularly uncommon, and localization in the cecum is exceptionally rare [3]. Undifferentiated sarcomas, defined by the absence of specific line of differentiation on histology and immunohistochemistry, constitute an aggressive and poorly characterized subgroup [5]. Due to the rarity of reported cases, diagnosis and management of this entity remain difficult [7].

Clinically, cecal undifferentiated sarcomas often present with nonspecific and misleading symptoms that closely resemble colorectal adenocarcinoma. Patients may report abdominal pain, altered bowel habits, anemia, gastrointestinal bleeding, weight loss, or a palpable mass. Because adenocarcinoma accounts for the vast majority of colorectal malignancies, it is usually the primary suspected diagnosis, which may delay consideration of a mesenchymal tumor [3,6].

Radiological evaluation plays a key role in detecting and staging the lesion. Contrast-enhanced CT typically reveals a large, heterogeneous mass, sometimes with areas of necrosis or hemorrhage. However, imaging findings are not pathognomonic and cannot reliably distinguish sarcomas from adenocarcinomas or other colonic neoplasms [5]. Magnetic resonance imaging (MRI) may provide additional information regarding local extension and tissue characteristics, while positron emission tomography (PET-CT) can assist in assessing metastatic spread. Definitive diagnosis ultimately relies on histopathological examination with immunohistochemical analysis to exclude other mesenchymal or epithelial tumors [9].

From a pathological standpoint, cecal undifferentiated sarcomas are characterized by high-grade pleomorphic spindle or epithelioid cells arranged in fascicles or sheets, with marked nuclear atypia and high mitotic activity [5]. Tumor necrosis is frequently observed. Immunohistochemistry is crucial to exclude other entities such as adenocarcinoma, GIST, lymphoma, or leiomyosarcoma. Typically, undifferentiated sarcomas lack specific lineage markers and may show variable vimentin positivity, while epithelial markers (cytokeratins), CD117, DOG1, desmin, and S100 are usually negative. Molecular studies, although not always performed, may help exclude specific translocation-associated sarcomas and refine classification, particularly in diagnostically challenging cases [10].

Complete surgical resection with negative margins remains the cornerstone of treatment. In our case, the deep surgical margin was positive, which is a major prognostic factor and may have contributed to the rapid disease progression. This underscores the importance of achieving negative margins whenever anatomically feasible. The role of adjuvant chemotherapy or radiotherapy is not clearly established and is often extrapolated from soft tissue sarcoma management protocols [11]. Metastatic undifferentiated sarcoma and unresectable sarcomas are associated with a poor prognosis [12]. In the palliative setting, therapeutic decisions are guided by the patient’s performance status, comorbidities, prior treatments, and symptom burden. Several chemotherapeutic agents and regimens have demonstrated activity in soft tissue sarcomas, including anthracycline-based combinations, gemcitabine-based regimens, and taxanes [13]. However, undifferentiated sarcoma is considered one of the most chemoresistant sarcoma subtypes. The role of chemotherapy remains unclear, and the available literature is limited due to the rarity of this tumor [12]. Pazopanib, a multi-kinase inhibitor, was evaluated in the phase III PALETTE (PAzopanib expLorEd in SofT-Tissue Sarcoma-a phasE III study) trial, which included patients with previously treated non-adipocytic soft tissue sarcomas. The study demonstrated an improvement in progression-free survival, without a significant impact on overall survival [14].

Given the aggressive nature of these tumors and the risk of recurrence or metastasis, close postoperative surveillance is mandatory. Reporting such rare cases contributes to improving awareness and understanding of this diagnostically challenging entity.

## Conclusions

Undifferentiated sarcomas of the digestive tract are rare and aggressive malignancies. Their unusual location may delay diagnosis and complicate management. Surgical resection remains the mainstay of treatment when feasible. However, advanced stages are frequently associated with limited therapeutic options. Overall survival remains significantly reduced in such cases. Further studies are needed to better define optimal therapeutic strategies and improve outcomes for this rare entity.
